# Combined Analysis of IFN-γ, IL-2, IL-5, IL-10, IL-1RA and MCP-1 in QFT Supernatant Is Useful for Distinguishing Active Tuberculosis from Latent Infection

**DOI:** 10.1371/journal.pone.0152483

**Published:** 2016-04-01

**Authors:** Maho Suzukawa, Shunsuke Akashi, Hideaki Nagai, Hiroyuki Nagase, Hiroyuki Nakamura, Hirotoshi Matsui, Akira Hebisawa, Ken Ohta

**Affiliations:** 1 National Hospital Organization Tokyo National Hospital, Tokyo, Japan; 2 Division of Respiratory Medicine and Allergology, Department of Medicine, Teikyo University School of Medicine, Tokyo, Japan; 3 Department of Environmental and Preventive Medicine, Graduate School of Medical Science, Kanazawa University, Ishikawa, Japan; University of Cape Town, SOUTH AFRICA

## Abstract

The QuantiFERON®-TB Gold In-Tube test (QFT), an interferon-γ release assay, is used to diagnose *Mycobacterium tuberculosis*, but its inaccuracy in distinguishing active tuberculosis from latent infection is a major concern. There is thus a need for an easy and accurate tool for achieving that goal in daily clinical settings. This study aimed to identify candidate cytokines for specifically differentiating active tuberculosis from latent infection. Our study population consisted of 31 active TB (tuberculosis) patients, 29 LTBI (latent tuberculosis infection) patients and 10 healthy control subjects. We assayed for 27 cytokines in QFT supernatants of both specific antigen-stimulated blood samples (TBAg) and negative-control samples (Nil). We analyzed their specificities and sensitivities by creating receiver operating characteristic (ROC) curves and measuring the area under those curves (AUCs). In TBAg–Nil supernatants, IL-10, IFN-γ, MCP-1 and IL-1RA showed high AUCs of 0.8120, 0.7842, 0.7419 and 0.7375, respectively. Compared with each cytokine alone, combined assay for these top four cytokines showed positive rates in diagnosing active TB, and GDA analysis revealed that MCP-1 and IL-5 are potent in distinguishing active TB from LTBI, with Wilk’s lambda = 0.718 (p < 0.001). Furthermore, utilizing the unique characteristic of IL-2 that its TBAg–Nil supernatant levels are higher in LTBI compared to active TB, the difference between IFN-γ and IL-2 showed a large AUC of 0.8910. In summary, besides IFN-γ, IL-2, IL-5, IL-10, IL-1RA and MCP-1 in QFT supernatants may be useful for distinguishing active TB from LTBI. Those cytokines may also help us understand the difference in pathogenesis between active TB and LTBI.

## Introduction

Tuberculosis is still among the most dangerous communicable infectious diseases in the world. Although the incidence of tuberculosis is slowly declining every year, WHO estimated that there were 9.0 million new cases of tuberculosis in 2013 [1]. Since interferon-γ release assays (IGRAs), including QuantiFERON®-TB Gold In-Tube test (QFT) (Cellestis Inc., Victoria, Australia), became readily available to clinical practitioners, diagnosis of *Mycobacterium tuberculosis* (*M*. *tuberculosis*) infection has become much easier and faster compared to when diagnosis relied on microbiologic methods such as mycobacterial culture, acid-fast smear and the Mantoux tuberculin skin test (TST).

Today, despite Japan’s high level of social health care, active tuberculosis is still seen. Another problem is that new cases are often (multi-)drug-resistant. Other complicating factors include increased prescription of immune-suppressive medications for specific diseases such as cancer and rheumatic diseases, increased numbers of immigrants and travelers from developing countries with a higher incidence of active TB, and increased prevalence of acquired immune disorders such as HIV infection. These multiple factors make diagnosis and management of *M*. *tuberculosis* infection even more complex and challenging than before [2, 3]. Thus, there is a need for more accurate, faster and easier diagnosis of *M*. *tuberculosis*, which would permit early treatment.

A profound concern of physicians with regard to IGRAs, including QFT, is their inability to discriminate active TB from LTBI [4, 5]. Moreover, QFT quite often gives a positive result for patients with a past history of tuberculosis infection, even if they received curative therapy for the disease. When a patient is QFT-positive, he/she is diagnosed as infected with tuberculosis and may be started on treatment even in the absence of other clinical data and symptoms. If the patient shows no other evidence of active TB, then he/she may be put on a single-drug regimen using isoniazid (INH) for at least 6 months, with monthly visits to the clinic. If the patient has no clinical symptoms, compliance may decrease due to psychological, economic or physical reasons [6]. In addition, daily use of INH may cause unnecessary side effects such as liver injury or allergic reactions, as well as select for drug-resistant mycobacteria [7]. Therefore, it would be useful to be able to identify other cytokines besides IFN-γ that could be measured in QFT supernatants, thereby increasing the sensitivity and specificity of QFT and making it easier to discriminate active TB from LTBI. Here, we report the results of our performance of multiplex cytokine analysis of QFT supernatants of samples from 31 patients with active TB and 29 patients with LTBI. We identified IL-2, IL-5, IL-10, IL-1RA and MCP-1 as new candidates to be measured in QFT supernatants for better differentiation of active TB from LTBI.

## Study Population and Methods

### Study Population

The protocol for this study was reviewed and approved by the National Hospital Organization Tokyo National Hospital Institutional Ethical Review Board (IRB). Informed verbal consent was obtained from all the study participants and documented in the medical records. The IRB approved this verbal informed consent procedure for this study because the participants needed to undergo QFT as part of their requisite clinical examinations or routine medical checkups, regardless of participation in this study, and leftover specimens were used for this study. The study population comprised 31 patients diagnosed as active TB, 29 patients with LTBI and 10 healthy control subjects. Patients and subjects who were examined by QFT at Tokyo National Hospital from February 2010 to December 2012 and were QFT-positive, 21 to 55 years of age, HIV-negative and not using immunosuppressive medications, and had no clinical complications, were consecutively enrolled in this study. The population was then further specified into active TB or LTBI. The control patients were examined by QFT as part of routine annual examinations of healthcare workers at Tokyo National Hospital. All the active TB and LTBI patients underwent QFT at the time of diagnosis, prior to initiation of therapy.

Active TB patients were defined as patients with abnormal radiologic findings suggestive of active pulmonary tuberculosis with microbiologic confirmation of infection with *M*. *tuberculosis* by mycobacterial culture, acid-fast smear examination and transcription reverse transcription concerted amplification (TRC) of sputa. All the active TB patients were untreated cases. LTBI is conventionally defined as presence of signs of infection with *M*. *tuberculosis* but with no evidence of active disease. In this study, the LTBI patients were QFT-positive, but had no clinical or physical findings, no symptoms of active TB and no abnormal chest X-ray findings. No sputum specimens were examined for LTBI or control subjects because they had almost no sputum. All TLBI and control subjects were selected from our hospital workers.

### QFT

QFT was performed according to the manufacturer’s instructions. Briefly, blood was drawn by venipuncture. Blood aliquots were then incubated at 37°C for 16–24 hours with either a mixture of ESAT-6, CFP-10 and TB7.7 as tuberculosis-specific antigens (TBAg) or a mitogen as a positive control, or without stimulation as a negative control (Nil). The culture supernatants were collected and used to quantitate IFN-γ by enzyme-linked immunosorbent assay using the QFT system. QFT was judged according to the manufacturer’s instructions.

### Multiple Cytokine Assay

Supernatants remaining from QFT were frozen at -20°C for as long as 5 years at Tokyo National Hospital and subsequently used for this study. The levels of cytokines in the TBAg supernatants and Nil supernatants were analyzed using a Bio-Plex Pro Human Cyokine Panel, 27-Plex (BioRad) and LUMINEX 200 (Luminex, Austin, TX) according to the manufacturers’ instructions. The analyzed cytokines were basic FGF, eotaxin, G-CSF, GM-CSF, IFN-γ, IL-1β, -1RA, -2, -4, -5, -6, -7, -8, -9, -10, -12, -13, -15 and -17A, IP-10, MCP-1, MIP-1α, MIP-1β, PDGF-BB, RANTES, TNF-α and VEGF. Prior to measuring the samples, the supernatants were diluted 4x according to the manufacturers’ instructions, or diluted 40x for measuring IL-8, IP-10, MCP-1, MIP-1α, MIP-1β and RANTES because those 6 cytokines were above the detection limit of Luminex kit when measured for 4x-diluted supernatants.

### Statistical Analysis

Continuous variables were expressed as medians with interquartile ranges. Overall comparisons between the three groups were done with 1-way ANOVA. Then *post hoc* Bonferroni comparisons were performed between the groups and *P* values were determined. *P* values of less than 0.05 were considered significant. We constructed receiver operating characteristic (ROC) curves, and the area under each ROC curve (AUC) was calculated.

We selected the top four cytokines based on their TBAg–Nil AUCs, i.e., IL-10, IFN-γ, MCP-1 and IL-1RA, and then we selected the cytokine value with the highest Youden Index as the cut-off value for the level of each cytokine in the supernatant. We assigned a score of 0 or 1 to each assay result depending on whether it was below or above the cut-off value for the cytokine. Then the sum of the four cytokine scores (total score) was calculated [8] and the percentages of active TB were calculated to see the accuracy of distinguishing active TB from LTBI.

Next, stepwise Wilk’s lambda discriminant analyses were performed as general discriminant analyses (GDA) to determine the candidate cytokines that contributed the most to the discrimination between active TB and LTBI. The stepwise procedures were guided by an F value probability of 0.05 for inclusion and 0.20 for exclusion. The coefficients for the cytokines included in the last step were calculated.

All statistical analyses were performed using GraphPad Prism version 5.0 (GraphPad Software, San Diego, CA) and SPSS version 23.0 (IBM, Armonk, NY).

## Results

### Study Subjects

All 70 enrolled subjects, consisting of 31 active TB patients, 29 LTBI patients and 10 healthy control subjects, were analyzed. Table 1 shows the demographic and clinical characteristics of all subjects. All the active TB patients had been diagnosed with pulmonary TB by pulmonologists on the basis of positive chest X-ray results and positive microbial examinations. We selected the active TB and LTBI patients from among QFT-positive subjects, and all the control subjects were QFT-negative. None of the LTBI or healthy control participants had comorbidities or a history of active TB. None of the participants were infected with HIV. The active TB and LTBI patients included more male patients and older patients compared to the healthy control subjects, but there was no statistical difference between the active TB and LTBI patients in regard to gender or age.

**Table 1:** Patient characteristics.

Group	All	Active	LTBI	Control	*p*-value
N (%)	70 (100)	31 (44)	29 (42)	10 (14)	
Male, N (%)	31 (45)	18 (58)	12 (41)	1 (10)	n.s.
Age (y) (range)	37 (21–55)	37 (21–48)	42 (23–55)	29 (25–35)	n.s.
Presence of TB history, N (%)	0 (0)	0 (0)	0 (0)	0 (0)	
QFT positive, N (%)	60 (86)	31 (100)	29 (100)	0 (0)	

p-value: active TB patients vs. LTBI patients.

### Differences in QFT supernatant cytokine levels between active TB and LTBI patients

#### TBAg–Nil supernatant

**Fig 1:**
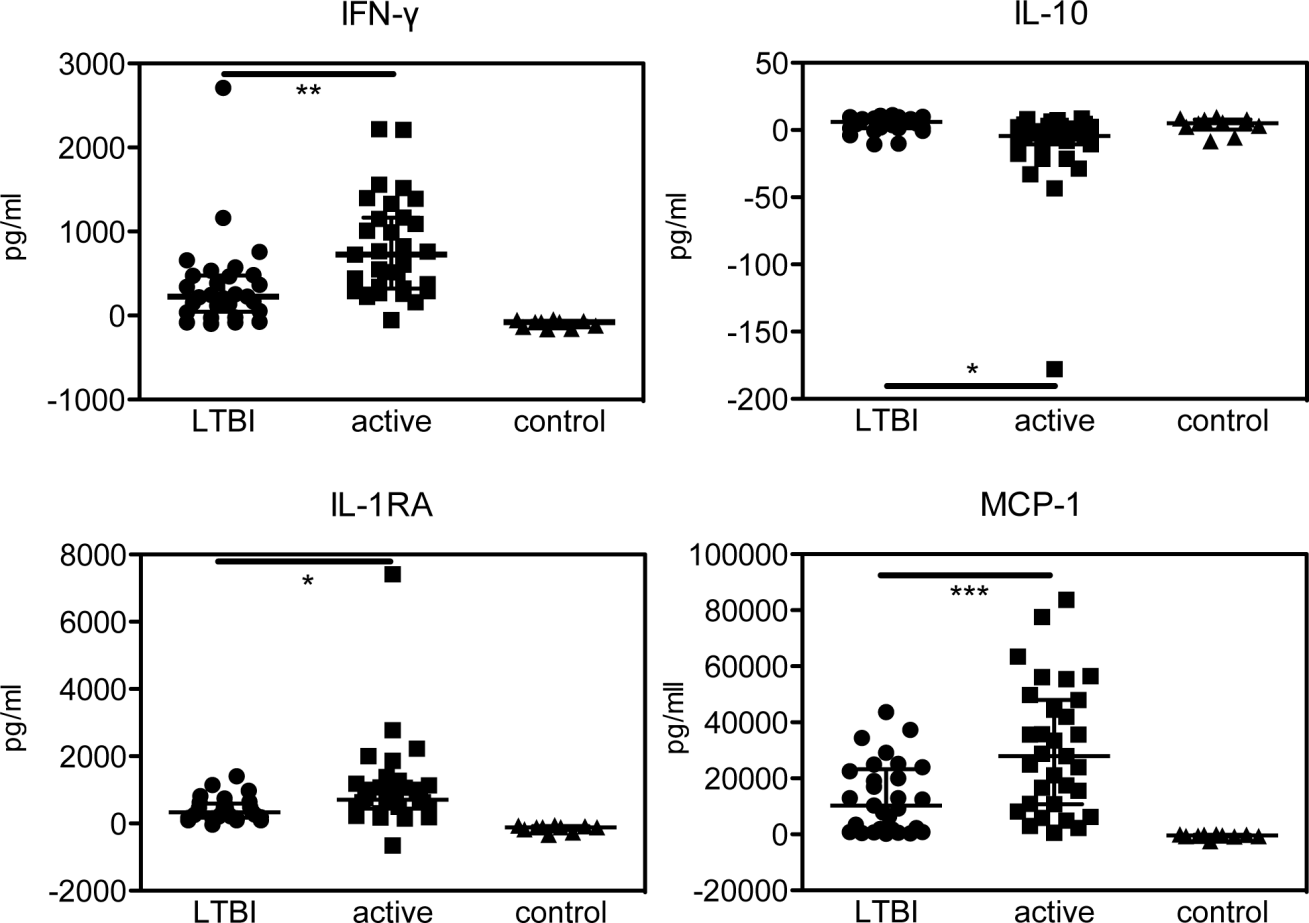
Major cytokines in TBAg–Nil supernatants of patients with active TB, LTBI and healthy controls. *** *P* < 0.001 and ** *P* < 0.01 between active TB vs. LTBI. Bars represent means, and error bars represent the SEM.

The cytokine levels in the QFT TBAg and Nil supernatants were measured by Luminex assay. Since QFT in the clinical setting is always judged on the basis of TBAg–Nil, we also determined that value (Table 2 and Fig 1). IFN-γ, IL-1RA, IL-8 and MCP-1 were significantly higher in the active TB patients compared to LTBI patients. Interestingly, IL-5 and IL-10 were significantly lower in the active TB patients compared to the LTBI patients, although the actual differences in their values are quite small. The TBAg–Nil data also found that several cytokines (IL-2, IP-10 and PDGF) showed a significant difference between the LTBI patients and the healthy control subjects.

**Table 2:** Concentrations of cytokines in the three groups (TBAg–Nil).

Cytokine	Median Concentration (IQR)			*p*-value		
	Active	LTBI	Control	Active vs LTBI	Active vs Control	LTBI vs Control
Basic FGF	-2.03 (-23.63–9.00)	-3.87 (-19.38–6.30)	-10.96 (-25.29–2.49)	N.S.	N.S.	N.S.
Eotaxin	-28.32 (-40.46–15.82)	-27.30 (-49.23–2.75)	-23.78 (-28.01–13.52)	N.S.	N.S.	N.S.
G-CSF	25.82 (-10.05–54.47)	-5.19 (-20.75–24.99)	27.52 (-15.04–45.74)	N.S.	N.S.	N.S.
GM-CSF	-15.50 (-30.80–7.80)	-16.65 (-32.20–5.64)	-31.83 (-44.24–7.24)	N.S.	N.S.	N.S.
IFN-γ	724.91 (321.78–1166.55)	224.73 (45.34–476.84)	-77.59 (-139.85–62.44)	0.003	<0.001	N.S.
IL-1β	-4.94 (-102.05–189.63)	4.21 (-97.46–80.62)	16.30 (-141.18–130.32)	N.S.	N.S.	N.S.
IL-1RA	707.23 (444.68–1184.28)	336.58 (176.53–592.04)	-117.08 (-199.78–78.61)	0.031	0.002	N.S.
IL-2	83.61 (43.71–185.37)	116.20 (23.44–311.57)	2.38 (-4.29–5.46)	N.S.	N.S.	0.047
IL-4	0.28 (-0.38–1.13)	0.53 (-0.12–1.83)	0.58 (-0.77–1.89)	N.S.	N.S.	N.S.
IL-5	-15.08 (-24.89–12.62)	-10.00 (-15.69–5.53)	-3.91 (-11.33–0.23)	0.014	0.01	N.S.
IL-6	176.55 (-48.81–1097.84)	31.12 (-96.44–738.96)	167.65 (65.80–951.83)	N.S.	N.S.	N.S.
IL-7	2.18 (-1.36–7.21)	2.18 (2.18–8.45)	4.66 (2.18–7.60)	N.S.	N.S.	N.S.
IL-8	20890.78 (7327.91–28423.52)	7415.39 (2365.61–15300.97)	13256.78 (6566.29–20347.51)	0.007	N.S.	N.S.
IL-9	-118.84 (-165.08–92.39)	-101.21 (-130.48–70.55)	-115.88 (-152.37–73.32)	N.S.	N.S.	N.S.
IL-10	-4.28 (-10.66–3.11)	6.07 (1.37–8.14)	5.15 (0.33–7.98)	0.023	N.S.	N.S.
IL-12	-11.01 (-27.53–3.68)	-0.29 (-9.47–9.71)	7.61 (1.45–10.06)	N.S.	N.S.	N.S.
IL-13	0.85 (-1.79–4.23)	-0.17 (-2.38–6.05)	-0.28 (-2.72–1.16)	N.S.	N.S.	N.S.
IL-15	-10.06 (-27.04–4.23)	1.55 (-9.55–1.55)	-1.92 (-9.94–1.55)	N.S.	N.S.	N.S.
IL-17A	-195.68 (-235.40–163.17)	-154.06 (-213.78–111.84)	-193.69 (-215.66–139.76)	N.S.	N.S.	N.S.
IP-10	52277.85 (31097.49–90807.68)	33045.90 (23854.53–71496.1)	559.11 (-382.88–1967.80)	N.S.	<0.001	0.009
MCP-1	27929.10 (10770.41–48038.45)	10299.09 (1293.56–23234.76)	-429.70 (-683.82–84.31)	0.001	<0.001	N.S.
MIP-1α	-382.65 (-882.75–76.53)	-150.39 (-793.64–37.93)	-302.47 (-825.80–137.43)	N.S.	N.S.	N.S.
MIP-1β	3250.81 (780.23–8292.59)	1520.92 (-82.31–6873.52)	1694.73 (1034.85–2106.29)	N.S.	N.S.	N.S.
PDGF-BB	3671.89 (2067.81–6657.97)	2443.54 (1195.44–4426.76)	-1600.84 (-1885.38–1314.11)	N.S.	<0.001	<0.001
RANTES	15836.36 (2136.57–35205.30)	440.27 (-20413.57–11500.78)	-30840.99 (-60681.70–14263.16)	N.S.	0.045	N.S.
TNF-α	-37.03 (-259.98–736.27)	-20.67 (-219.56–94.27)	-497.81 (-819.28–300.54)	N.S.	0.032	N.S.
VEGF	2.56 (-25.07–41.47)	0.93 (-8.45–17.91)	57.39 (18.15–93.38)	N.S.	N.S.	N.S.

#### Nil supernatant

Unlike the case of TBAg–Nil (Table 2 and Fig 1), Nil supernatants showed bigger differences between active TB and LTBI patients in terms of the number of cytokines that showed statistical significance (S1 Table and S1 Fig). Interestingly even in the Nil supernatants, many of the cytokines were significantly increased in the active TB patients compared to LTBI patients (S1 Table and S1 Fig). Among the 25 cytokines tested, Basic FGF, G-CSF, IFN-γ, IL-1β, IL-1RA, IL-2, -4, -5, -10, -12, -13, -15, -17A, MCP-1, PDGF, TNF-α and VEGF were significantly elevated in the active TB patients compared to the LTBI patients. On the other hand, none of the cytokines showed a significant difference between the LTBI patients and the healthy control subjects.

### Accuracy of each cytokine marker in differentiating between active TB and LTBI

#### TBAg–Nil supernatant

**Fig 2:**
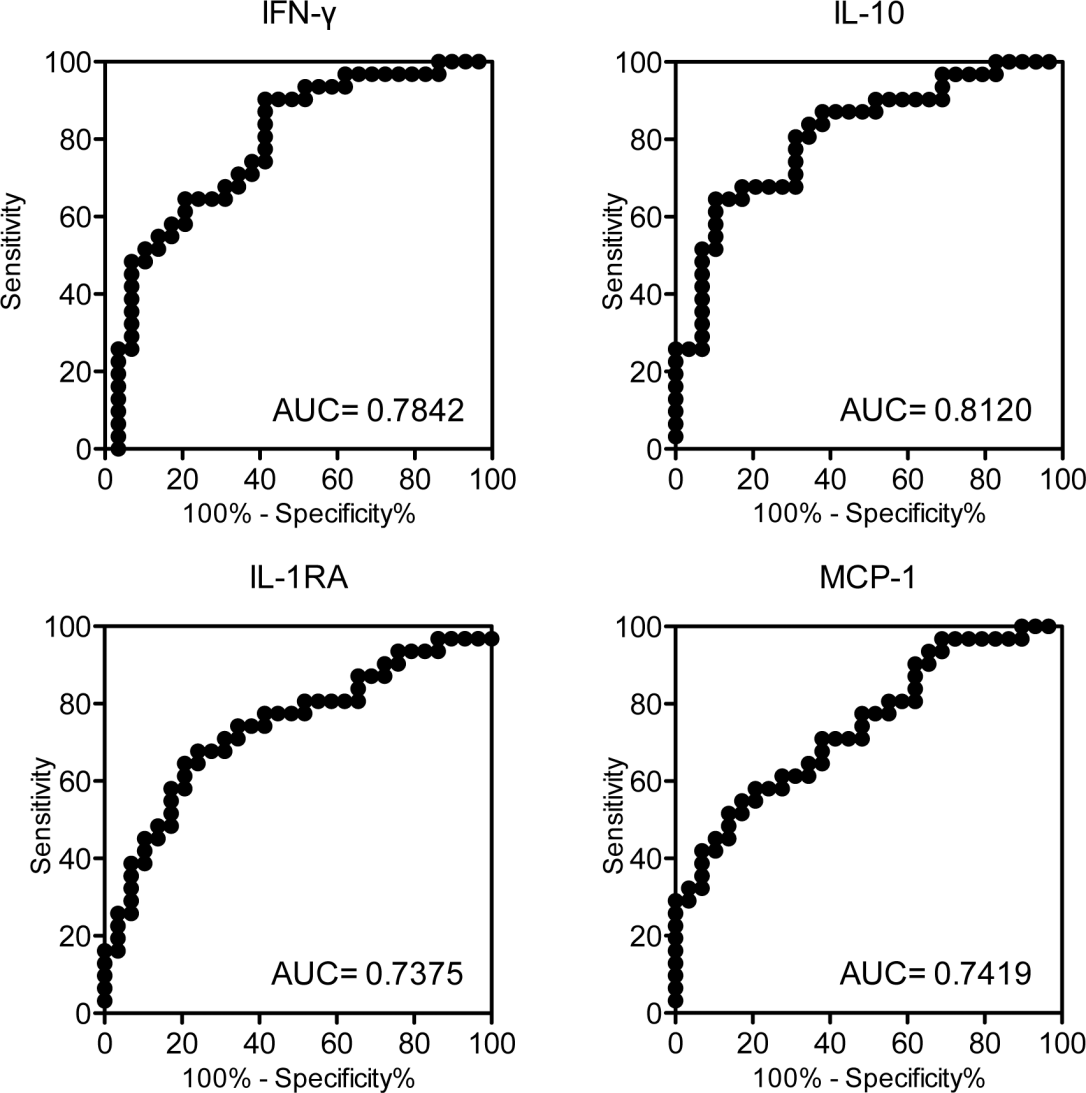
Major ROC curves comparing the diagnostic accuracy of cytokines in TBAg–Nil supernatants for differentiating active TB from LTBI. AUCs for each cytokine are shown in the graph.

To elucidate the accuracy of these markers in diagnosing active TB, ROC curves were created, and their AUCs were calculated. Sensitivities and specificities were also calculated using the value with the highest Youden Index as the cut-off value (Table 3 and S2 Table). The highest AUCs obtained for TBAg–Nil supernatants were shown by IL-10, IFN-γ, MCP-1 and IL-1RA (Fig 2). Several other cytokines, i.e., IL-5, -8, -12, -15, -17A, PDGF and RANTES, also showed statistical significance in differentiating active TB from LTBI (Table 3).

**Table 3:** AUCs for discriminating active tuberculosis from LTBI (TBAg–Nil).

Cytokine	AUC (95% CI)	*p*-value	Cut-off	Sensitivity, % (95% CI)	Specificity, % (95% CI)
Basic FGF	0.50 (0.35–0.65)	N.S.	-2.2	51.61 (33.06–69.85)	58.62 (38.94–76.48)
Eotaxin	0.51 (0.35–0.66)	N.S.	-14.9	80.65 (62.53–92.55)	41.38 (23.52–61.06)
G-CSF	0.62 (0.48–0.77)	N.S.	14.7	61.29 (42.19–78.15)	72.41 (52.76–87.27)
GM-CSF	0.56 (0.42–0.71)	N.S.	-2.7	38.71 (21.85–57.81)	82.76 (64.23–94.15)
IFN-γ	0.78 (0.67–0.90)	<0.001	256.9	90.32 (74.25–97.96)	58.62 (38.94–76.48)
IL-1β	0.51 (0.36–0.66)	N.S.	179.4	25.81 (11.86–44.61)	93.1 (77.23–99.15)
IL-1RA	0.74 (0.61–0.86)	0.002	631.9	64.52 (45.37–80.77)	79.31 (60.28–92.01)
IL-2	0.55 (0.40–0.70)	N.S.	333.2	96.77 (83.30–99.92)	24.14 (10.30–43.54)
IL-4	0.58 (0.44–0.73)	N.S.	0.3	51.61 (33.06–69.85)	68.97 (49.17–84.72)
IL-5	0.70 (0.57–0.83)	0.007	-11.3	87.1 (70.17–96.37)	51.72 (32.53–70.55)
IL-6	0.54 (0.39–0.69)	N.S.	53.2	61.29 (42.19–78.15)	55.17 (35.69–73.55)
IL-7	0.60 (0.45–0.74)	N.S.	5.2	70.97 (51.96–85.78)	48.28 (29.45–67.47)
IL-8	0.71 (0.58–0.85)	0.005	16088.0	66.67 (47.19–82.71)	82.76 (64.23–94.15)
IL-9	0.6352 (0.493–0.78)	N.S.	-154.1	32.26 (16.68–51.37)	93.1 (77.23–99.15)
IL-10	0.81 (0.70–0.92)	<0.001	-0.8	64.52 (45.37–80.77)	89.66 (72.65–97.81)
IL-12	0.70 (0.56–0.83)	0.009	-10.3	54.84 (36.03–72.68)	79.31 (60.28–92.01)
IL-13	0.51 (0.36–0.66)	N.S.	0.1	54.84 (36.03–72.68)	55.17 (35.69–73.55)
IL-15	0.73 (0.60–0.86)	0.002	0.3	83.87 (66.27–94.55)	65.52 (45.67–82.06)
IL-17	0.66 (0.52–0.80)	0.030	-155.3	87.1 (70.17–96.37)	51.72 (32.53–70.55)
IP-10	0.62 (0.48–0.76)	N.S.	33082.0	73.33 (54.11–87.72)	51.72 (32.53–70.55)
MCP-1	0.74 (0.62–0.87)	0.001	26573.0	51.61 (33.06–69.85)	86.21 (68.34–96.11)
MIP-1α	0.60 (0.46–0.75)	N.S.	-300.3	60.0 (40.60–77.34)	68.97 (49.17–84.72)
MIP-1β	0.57 (0.42–0.72)	N.S.	1686.0	70.0 (50.60–85.27)	51.72 (32.53–70.55)
PDGF-BB	0.65 (0.51–0.79)	0.042	1516.0	90.32 (74.25–97.96)	37.93 (20.69–57.74)
RANTES	0.69 (0.56–0.83)	0.011	13836.0	53.33 (34.33–71.66)	79.31 (60.28–92.01)
TNF-α	0.53 (0.38–0.68)	N.S.	660.6	29.03 (14.22–48.04)	93.1 (77.23–99.15)
VEGF	0.50 (0.35–0.65)	N.S.	-23.4	25.81 (11.86–44.61)	93.1 (77.23–99.15)

95% CI = 95% confidence interval.

#### Nil supernatant

On the other hand, in the Nil supernatants, IL-1RA, MCP-1, IL-15, IL-12 and IL-10 showed higher AUCs than IFN-γ (S2 Table and S2 Fig). Many of the other cytokines also showed high AUCs, with statistical significance in discriminating active TB from LTBI (S2 Table and S2 Fig).

### Accuracy of cytokine combinations in differentiating between active TB and LTBI

**Fig 3:**
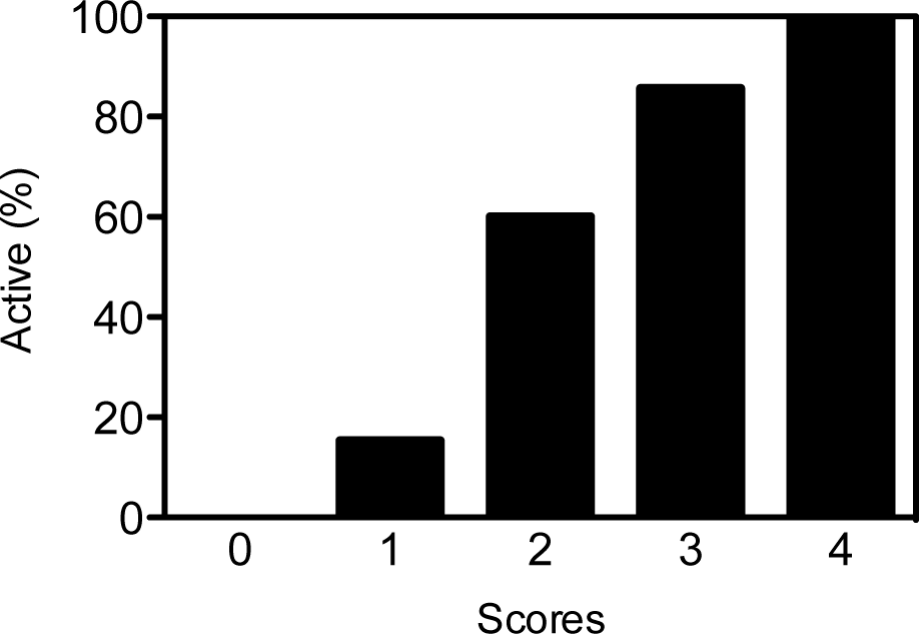
Rates of identification of active TB on the basis of the total score for combination of four cytokines (IL-10, IFN-γ, MCP-1 and IL-1RA) in TBAg–Nil supernatant.

Next, combinations of multiple cytokine markers were examined to see if that would improve the accuracy in differentiating active TB from LTBI. For the combinations, we chose the four best cytokines based on their TBAg–Nil AUCs, namely, IL-10, IFN-γ, MCP-1 and IL-1RA (Table 3). As shown in Fig 3, the rate of identification of active TB increased with the total score. The total score of 4 for TBAg–Nil supernatants showed 100% identification of active TB (Fig 3).

### GDA analysis of cytokine combinations in differentiating between active TB and LTBI

To test the accuracy of cytokine combinations in differentiating between active TB and LTBI, we performed GDA analysis using TBAg–Nil supernatants. We selected age, sex, IFN-γ, IL-1RA, -5, -8, -10, -17A, MCP-1 and PDGF as factors, and performed GDA analysis using Wilk’s lambda. The final stepwise analysis selected IL-5 and MCP-1 with Wilk’s lambda = 0.718 (p < 0.001), and the coefficients were -0.655 for IL-5 and 0.821 for MCP-1.

### Accuracy of cytokine ratios and cytokine differences for differential diagnosis of active TB and LTBI

**Fig 4:**
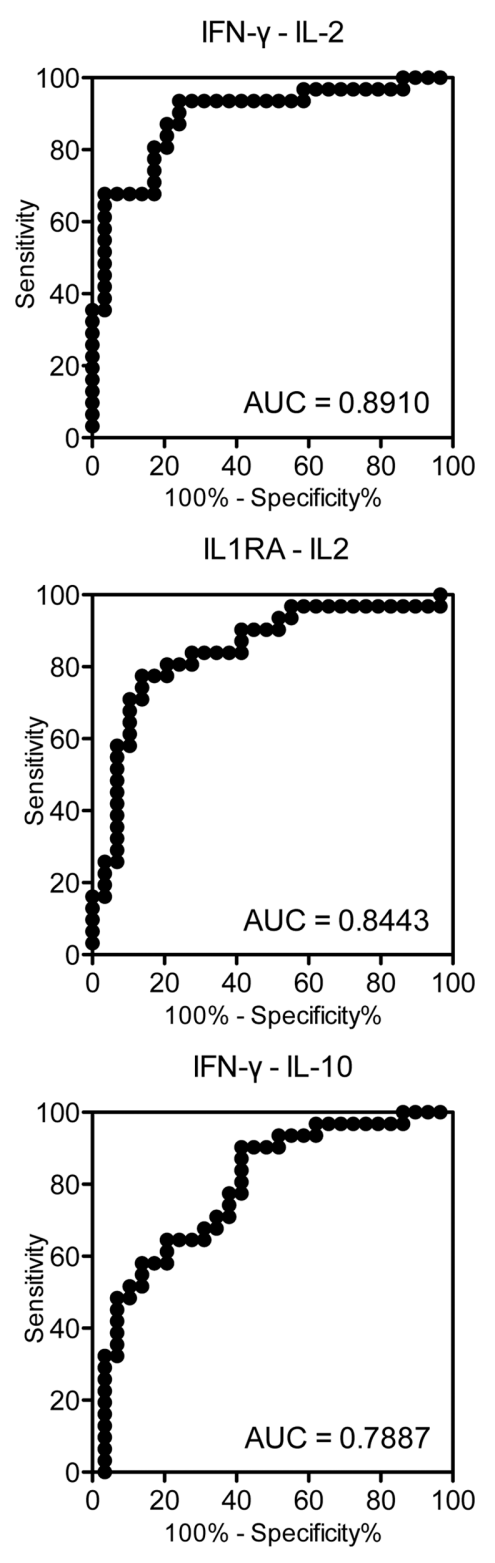
ROC curves comparing the diagnostic accuracy of differences between two cytokines in TBAg–Nil supernatants for differentiating active TB from LTBI. The AUCs are shown.

The levels of several cytokines, i.e., IL-2, -5, -10 and -15, were higher in TBAg–Nil supernatants of LTBI patients compared with active TB patients, which is the opposite tendency from the other cytokines. For that reason, we calculated the ratios and differences of those cytokines relative to IFN-γ, IL-1RA and MCP-1. As a result, larger AUCs were shown by the difference between two cytokines than by their ratio. Our data show a larger AUC for the difference (0.8910) between IFN-γ and IL-2 compared with for their ratio (0.7164). The ratios of the other pairs of cytokines did not show AUCs above 0.8, but their differences by subtraction showed large AUCs, such as 0.8443 for IL-1RA–IL-2 (Fig 4).

## Discussion

We found that several cytokines in Nil as well as TBAg-stimulated QFT supernatants were useful in differentiating active TB from LTBI. Although IFN-γ is considered to be unable to distinguish active TB from LTBI, our present study found that IL-1RA, IL-2, IL-5, MCP-1 and IL-10—in addition to IFN-γ—are good candidates; especially when analyzed in combination, they increase the diagnostic potential of QFT for discriminating active TB from LTBI.

At present, the most specific immunoassays for diagnosing mycobacterial infections are probably IGRAs, including QFT. However, a problem in using IGRAs is that they are unable to discriminate active TB from LTBI. Indeed, for smear-negative patients, QFT has been estimated to show sensitivity of 75% and specificity of only 37%, suggesting that the diagnostic accuracy of QFT is especially low in those patients [9]. TST is also widely used in diagnostic testing for LTBI [10], but TST has low sensitivity of 80% [11] in subjects who had been vaccinated with BCG, which is common in Japan. Therefore, our aim here was to elucidate if we could improve diagnosis of active TB simply by assaying for a larger number of cytokines in the QFT supernatant used to detect IFN-γ. The major advantages in utilizing the QFT supernatant for differential diagnosis of active TB are its microbial specificity and methodological convenience for direct assay of cytokines induced by the TBAg-stimulation. The ability to discriminate active TB from LTBI by measuring multiple cytokines in a small amount of blood in an overnight assay would be a big advantage over the currently available examinations, including microbial culture.

Several cytokines showed a significant difference between active TB and LTBI in the TBAg–Nil supernatant. Interestingly, some of these cytokines showed the reverse pattern in TBAg–Nil supernatants between active TB and LTBI, i.e., higher in LTBI compared to active TB. In line with another study showing that TBAg-stimulated IL-10 is low in active TB [12], we found that IL-10 had the best AUC for discriminating active TB from LTBI, being higher in LTBI than in active TB. IL-10 is produced by various hematopoietic cells, and its main role is to suppress macrophage and dendritic cell functions [13]. IL-10 has also been reported to inhibit formation of mature fibrotic granuloma during *M*. *tuberculosis* infection [14]. We and others [12] showed that lymphocytes from LTBI patients produce more IL-10 in response to *in vitro* TBAg exposure, suggesting that LTBI lymphocytes may contribute to attenuating inflammation during *M*. *tuberculosis* infection.

Other cytokines that showed good AUCs in distinguishing active TB from LTBI were MCP-1 and IL-1RA, i.e., higher in active TB compared to LTBI. MCP-1 induces chemotaxis of monocytes and granulocytes, a function that seems critical for protection against microbial infection [15]. The fact that a single nucleotide polymorphism (SNP) in the MCP-1 promoter correlated with increased susceptibility to active TB disease [16] suggests a close relationship between MCP-1 and the pathogenesis of active TB. On the other hand, IL-1RA is secreted by monocytes, neutrophils and such structural cells as epithelial cells, and its role is competitive inhibition of the proinflammatory effects of IL-1α and IL-1β [17, 18]. IL-1RA has been suggested as a plasma biomarker in many inflammatory and infectious diseases, including TB [15]. Studies have shown that IL-1RA is significantly increased in the serum [19], BAL fluid [20] and QFT supernatant [21] in active TB. According to our present data and reports from other groups showing the importance of IL-1RA in differentiating active TB from LTBI in children [22, 23], IL-1RA may be a critical player or a by-product in the pathogenesis of active TB. However, more detailed examinations are needed of the physiological functions and roles of IL-1RA and MCP-1 in both active TB and LTBI.

It is noteworthy that Nil supernatants (i.e., without TBAg stimulation) showed bigger differences in cytokine levels between active TB and LTBI than found for TBAg–Nil and showed the highest AUCs. The primary reason for this is probably that active TB cases had greater systemic inflammation compared to LTBI. However, another reason may be that non-blood cells that are not used for QFT measurement also produce the assayed cytokines, resulting in the differences in cytokine levels being larger in Nil supernatants than in TBAg-stimulated supernatants. Indeed, MCP-1 and IL-1RA are produced by fibroblasts as well as by such blood cells as monocytes, macrophages and neutrophils [24, 25, 26]. Since only blood cells are used for QFT, there may not be significant cytokine induction in response to an antigen. Moreover, lymphocytes and monocytes from active TB patients may have already been maximally stimulated by antigen *in vivo*, such that their cytokine synthesis cannot be further increased *in vitro*, and resulting in differences in TBAg-Nil supernatants that are insufficient for discriminating between active TB and LTBI. It is also important that the levels of both MCP-1 and IL-1RA in Nil supernatants did not correlate positively with other clinical data related to inflammation (e.g., WBC, CRP, ESR) in our study (S3 Table). Thus, these cytokines may be independently and uniquely useful for differential diagnosis of active TB and LTBI, rather than being elevated due to a pro-inflammatory state.

When we analyzed combinations of multiple cytokines for their ability to discriminate active TB from LTBI, TBAg–Nil supernatants showed good results. Not only the combinations of four cytokines showed accurate diagnosis of active TB: our GDA analysis showed combination of MCP-1 and IL-5 may also be a good candidate for discriminating active TB from LTBI. Another study of analysis of combinations of multiple cytokines in unstimulated plasma for distinguishing active TB from household contacts (QFT-positive and negative) found that the best model was a combination of fractalkine, IFN-γ, IL-4, IL-10 and TNF-α [27]. Others found that combination of EGF, sCD40L, VEGF, TGF-α and IL-1α was potent for discriminating active TB and LTBI [28]. Together, their and our data indicate that combinations of several cytokines may provide clearer identification of active TB from LTBI than a single cytokine assay. However, larger, prospective studies are still necessary to identify the best combination.

It has already been reported that IL-2 was higher in LTBI compared to active TB [29, 30] (although our present study found only a tendency, without statistical significance). For that reason, IL-2/IFN-γ has been reported to be a useful value for differentiating active TB from LTBI [29]. Our further analyses using IL-2 and IL-10—two cytokines that were more elevated in LTBI patients than in active TB—on the basis of their differences and ratios relative to other cytokines indicated that these cytokines can be additional useful markers for discriminating active TB from LTBI.

Limitations of the present study include the relatively small numbers of patients with LTBI and healthy control subjects who all worked in healthcare and had unknown histories with regard to old TB and BCG vaccinations. In addition, the samples had been collected and kept frozen for some time. However, our study found a robust IFN-γ response to TBAg that agreed with the results of QFT, suggesting that there was no deterioration of the samples or technical error. Although there were differences in age among the subject groups, an earlier study found minimal differences in cytokine levels among different age groups [31]. Another limitation of our present study is the lack of sick control patients or a disease control. The patients in our study had been diagnosed only with *M*. *tuberculosis*. Therefore, we cannot affirm that the elevated cytokine levels we observed in our study population were *M*. *tuberculosis*-specific. They may have been a non-specific phenomenon observed in general inflammatory conditions, including infection with other microorganism(s) [32]. In the future, larger, prospective studies are needed to identify the optimal combinations of cytokines, confirm the clinical utility of assay of them as diagnostic markers of mycobacterial infection, especially for differentiating active TB from latent infection, and also to confirm their cut-off values. Understanding the cytokines that differentiate active TB from LTBI may help us elucidate the differences in pathogenesis between active and latent infections.

## Supporting Information

S1 Fig Top four cytokines in Nil supernatants of patients with active TB, LTBI and healthy controls. (PPTX)

S2 Fig Top four ROC curves comparing the diagnostic accuracy of cytokines in Nil supernatants for differentiating active TB from LTBI. (PPTX)

S1 Table Concentrations of cytokines in the three groups (Nil). (DOCX)

S2 Table AUCs for discriminating active tuberculosis from LTBI (Nil). (DOCX)

S3 Table Correlations between concentrations of cytokines and clinical findings (Nil). (DOCX)
