# Supplementary material for: Combined Analysis of IFN-γ, IL-2, IL-5, IL-10, IL-1RA and MCP-1 in QFT Supernatant Is Useful for Distinguishing Active Tuberculosis from Latent Infection
Source: PLoS One. 2016 Apr 1;11(4):e0152483. doi: 10.1371/journal.pone.0152483 (PMC4817970; doi:10.1371/journal.pone.0152483)
Supplement: S1 Fig — (PPTX) [file pone.0152483.s001.pptx]

## Slide 1
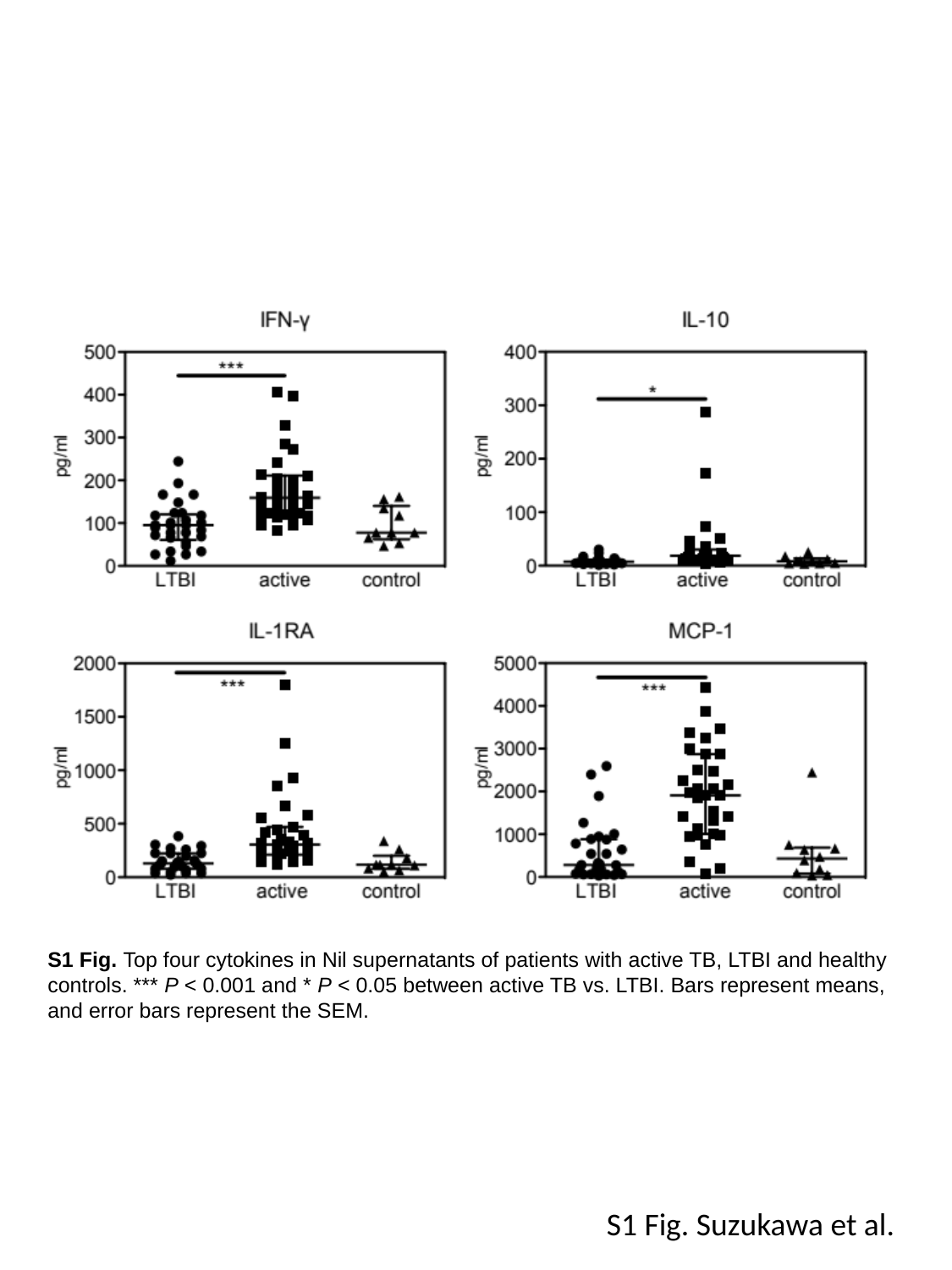

S1 Fig. Top four cytokines in Nil supernatants of patients with active TB, LTBI and healthy controls. *** P < 0.001 and * P < 0.05 between active TB vs. LTBI. Bars represent means, and error bars represent the SEM.
S1 Fig. Suzukawa et al.
