# Supplementary material for: Combined Analysis of IFN-γ, IL-2, IL-5, IL-10, IL-1RA and MCP-1 in QFT Supernatant Is Useful for Distinguishing Active Tuberculosis from Latent Infection
Source: PLoS One. 2016 Apr 1;11(4):e0152483. doi: 10.1371/journal.pone.0152483 (PMC4817970; doi:10.1371/journal.pone.0152483)
Supplement: S2 Fig — (PPTX) [file pone.0152483.s002.pptx]

## Slide 1
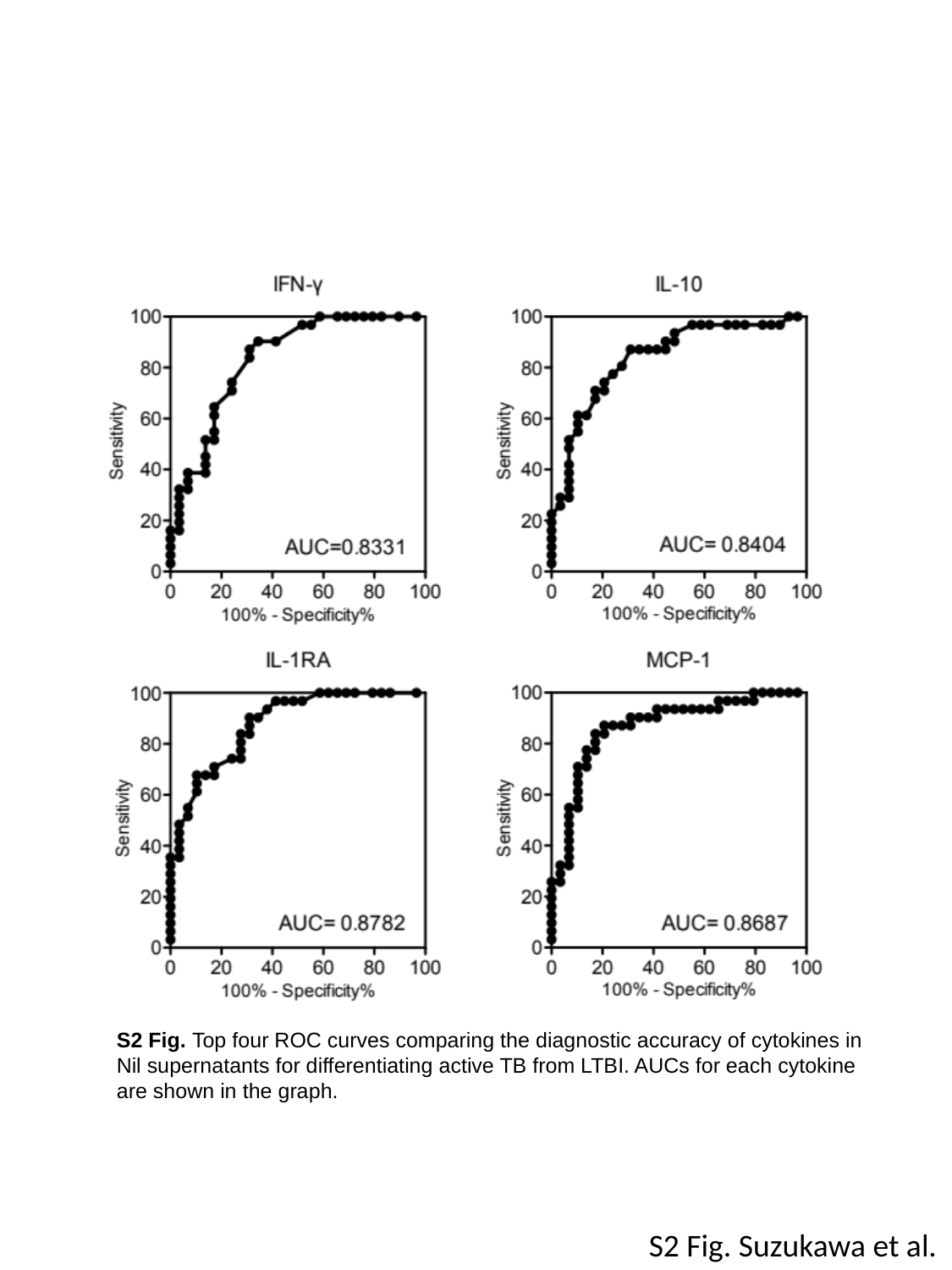

S2 Fig. Top four ROC curves comparing the diagnostic accuracy of cytokines in Nil supernatants for differentiating active TB from LTBI. AUCs for each cytokine are shown in the graph.
S2 Fig. Suzukawa et al.
