# Supplementary material for: Combined Analysis of IFN-γ, IL-2, IL-5, IL-10, IL-1RA and MCP-1 in QFT Supernatant Is Useful for Distinguishing Active Tuberculosis from Latent Infection
Source: PLoS One. 2016 Apr 1;11(4):e0152483. doi: 10.1371/journal.pone.0152483 (PMC4817970; doi:10.1371/journal.pone.0152483)
Supplement: S1 Table — (DOCX) [file pone.0152483.s003.docx]

S1 Table. Concentrations of cytokines in the three groups (Nil).

| Cytokine | Median Concentration (IQR) | | | *p*-value | | |
| --- | --- | --- | --- | --- | --- | --- |
|  | Active | LTBI | Control | Active vs LTBI | Active vs Control | LTBI vs Control |
| Basic FGF | 87.94 (76.08-99.76) | 69.86 (51.44-82.685) | 71.105 (66.03-75.16) | 0.003 | N.S. | N.S. |
| Eotaxin | 83.92 (66.82-102.93) | 64.33 (47.41-92.29) | 58.48 (41.97-70.52) | N.S. | 0.031 | N.S. |
| G-CSF | 71.75 (47.06-115.72) | 32.91 (22.63-57.48) | 40.58 (18.00-70.61) | 0.004 | 0.031 | N.S. |
| GM-CSF | 95.29 (61.94-137.22) | 79.55 (63.35-102.05) | 86.145 (29.97-125.76) | N.S. | N.S. | N.S. |
| IFN-γ | 159 (117.99-211.32) | 95.25 (60.92-120.78) | 77.64 (62.45-140.0) | <0.001 | 0.004 | N.S. |
| IL-1β | 231.83 (162.42-576.87) | 109.33 (18.13-264.32) | 194.105 (152.67-247.79) | 0.046 | N.S. | N.S. |
| IL-1RA | 304.89 (210.67-471.82) | 128.54 (78.04-223.48) | 117.28 (78.75-200.07) | <0.001 | 0.007 | N.S. |
| IL-2 | 17.38 (10.13-24.64) | 7.64 (4.04-14.39) | 9.88 (3.775-16.185) | 0.007 | N.S. | N.S. |
| IL-4 | 5.04 (4.14-6.33) | 3.27 (2.15-4.6) | 3.05 (2.15-4.77) | 0.001 | 0.022 | N.S. |
| IL-5 | 17.42 (14.96-27.23) | 12.34 (7.87-18.59) | 6.25 (2.11-13.67) | 0.041 | 0.038 | N.S. |
| IL-6 | 641.8 (226.74-1511.09) | 219.23 (48.51-1287.96) | 380.325 (107.93-1142.07) | N.S. | N.S. | N.S. |
| IL-7 | 3.64 (0.1-6.68) | 0.1 (0.1-2.355) | 0.1 (0.1-0.1) | N.S. | N.S. | N.S. |
| IL-8 | 6897.75 (3171.94-10539.89) | 2232.25 (1323.53-7973.18) | 3796.48 (2802.54-6311.98) | N.S. | N.S. | N.S. |
| IL-9 | 215.21 (196.09-248.88) | 180.23 (151.6-226.14) | 182.37 (138.02-246.05) | N.S. | N.S. | N.S. |
| IL-10 | 18.48 (11.48-30.05) | 7.22 (4.64-11.23) | 8.315 (4.41-13.68) | 0.037 | N.S. | N.S. |
| IL-12 | 56.13 (31.59-71.44) | 21.53 (15.3-37.5) | 29.04 (22.42-30.50) | <0.001 | 0.016 | N.S. |
| IL-13 | 7.71 (6.17-11.22) | 4.59 (2.96-7.71) | 3.235 (2.82-5.12) | 0.046 | N.S. | N.S. |
| IL-15 | 30.52 (20.89-46.31) | 0.14 (0.14-11.24) | 3.61 (0.14-11.63) | <0.001 | <0.001 | N.S. |
| IL-17A | 251.02 (219.26-292.36) | 210.68 (160.21-258.34) | 236.99 (172.56-261.68) | 0.003 | N.S. | N.S. |
| IP-10 | 4310.88 (2044.42-10708.71) | 2942.51 (1595.925-6275.31) | 1439.375 (733.94-5154.24) | N.S. | N.S. | N.S. |
| MCP-1 | 1909.51 (1006.23-2872.73) | 281.17 (78.425-881.37) | 431.96 (84.99-687.78) | <0.001 | <0.001 | N.S. |
| MIP-1α | 382.74 (76.58-884.18) | 150.46 (38.01-795.65) | 303.295 (138.10-827.35) | N.S. | N.S. | N.S. |
| MIP-1β | 3596.25 (1553.21-6430.99) | 2735.98 (1533.435-6713.575) | 3634.205 (1189.35-6384.20) | N.S. | N.S. | N.S. |
| PDGF-BB | 2837.26 (1965-3991.9) | 1959.58 (872.12-2568.47) | 1606.61 (1317.9-1890.13) | <0.001 | 0.006 | N.S. |
| RANTES | 51698.09 (33941.99-76771.48) | 44146.19 (31924.11-61889.47) | 30887.4 (14297.59-60740.05) | N.S. | N.S. | N.S. |
| TNF-α | 536.75 (326.94-1858.34) | 147.46 (69.48-440.34) | 498.52 (301.21-820.22) | 0.006 | N.S. | N.S. |
| VEGF | 89.78 (56.86-158.41) | 34.36 (21.73-69.66) | 41.04 (36.65-55.49) | <0.001 | 0.019 | N.S. |
