# Supplementary material for: Combined Analysis of IFN-γ, IL-2, IL-5, IL-10, IL-1RA and MCP-1 in QFT Supernatant Is Useful for Distinguishing Active Tuberculosis from Latent Infection
Source: PLoS One. 2016 Apr 1;11(4):e0152483. doi: 10.1371/journal.pone.0152483 (PMC4817970; doi:10.1371/journal.pone.0152483)
Supplement: S2 Table — (DOCX) [file pone.0152483.s004.docx]

S2 Table. AUCs for discriminating active tuberculosis from LTBI (Nil).

| Cytokine | AUC (95% CI) | *p*-value | Cut-off | Sensitivity, % (95% CI) | Specificity, % (95% CI) |
| --- | --- | --- | --- | --- | --- |
| Basic FGF | 0.75 (0.62-0.88) | 0.001 | 73 | 83.87 (66.27-94.55) | 62.07 (42.26-79.31) |
| Eotaxin | 0.65 (0.51-0.79) | 0.048 | 65.6 | 77.42 (58.90-90.41) | 51.72 (32.53-70.55) |
| G-CSF | 0.76 (0.64-0.88) | <0.001 | 59.8 | 61.29 (42.19-78.15) | 79.31 (60.28-92.01) |
| GM-CSF | 0.54 (0.39-0.69) | N.S. | 104.9 | 45.16 (27.32-63.97) | 79.31 (60.28-92.01) |
| IFN-γ | 0.83 (0.73-0.94) | <0.001 | 111 | 87.1 (70.17-96.37) | 68.97 (49.17-84.72) |
| IL-1β | 0.72 (0.59-0.85) | 0.003 | 160.2 | 77.42 (58.90-90.41) | 68.97 (49.17-84.72) |
| IL-1RA | 0.88 (0.79-0.96) | <0.001 | 158.2 | 90.32 (74.25-97.96) | 68.97 (49.17-84.72) |
| IL-2 | 0.76 (0.64-0.88) | <0.001 | 13.4 | 70.97 (51.96-85.78) | 75.86 (56.46-89.70) |
| IL-4 | 0.77 (0.65-0.89) | <0.001 | 3.6 | 87.1 (70.17-96.37) | 62.07 (42.26-79.31) |
| IL-5 | 0.69 (0.56-0.83) | 0.010 | 13.7 | 87.1 (70.17-96.37) | 51.72 (32.53-70.55) |
| IL-6 | 0.64 (0.50-0.78) | N.S. | 221.7 | 83.87 (66.27-94.55) | 51.72 (32.53-70.55) |
| IL-7 | 0.72 (0.58-0.85) | 0.004 | 1.4 | 70.97 (51.96-85.78) | 72.41 (52.76-87.27) |
| IL-8 | 0.66 (0.51-0.80) | 0.039 | 2863 | 80.65 (62.53-92.55) | 58.62 (38.94-76.48) |
| IL-9 | 0.68 (0.54-0.81) | 0.020 | 188.4 | 80.65 (62.53-92.55) | 62.07 (42.26-79.31) |
| IL-10 | 0.84 (0.74-0.94) | <0.001 | 10.6 | 87.1 (70.17-96.37) | 68.97 (49.17-84.72) |
| IL-12 | 0.85 (0.75-0.94) | <0.001 | 25.4 | 93.55 (78.58-99.21) | 65.52 (45.67-82.06) |
| IL-13 | 0.78 (0.66-0.90) | <0.001 | 6.4 | 74.19 (55.39-88.14) | 72.41 (52.76-87.27) |
| IL-15 | 0.86 (0.77-0.96) | <0.001 | 17.1 | 77.42 (58.90-90.41) | 82.76 (64.23-94.15) |
| IL-17A | 0.74 (0.61-0.86) | 0.002 | 237.6 | 67.74 (48.63-83.32) | 72.41 (52.76-87.27) |
| IP-10 | 0.62 (0.47-0.76) | N.S. | 3669 | 64.52 (45.37-80.77) | 65.52 (45.67-82.06) |
| MCP-1 | 0.87 (0.78-0.96) | <0.001 | 958.8 | 83.87 (66.27-94.55) | 82.76 (64.23-94.15) |
| MIP-1α | 0.59 (0.44-0.73) | N.S. | 300.7 | 58.06 (39.08-75.45) | 68.97 (49.17-84.72) |
| MIP-1β | 0.53 (0.39-0.68) | N.S. | 3004 | 64.52 (45.37-80.77) | 55.17 (35.69-73.55) |
| PDGF-BB | 0.74 (0.62-0.87) | 0.001 | 2866 | 48.39 (30.15-66.94) | 93.1 (77.23-99.15) |
| RANTES | 0.55 (0.40-0.69) | N.S. | 49214 | 61.29 (42.19-78.15) | 55.17 (35.69-73.55) |
| TNF-α | 0.77 (0.65-0.89) | <0.001 | 233.5 | 87.1 (70.17-96.37) | 65.52 (45.67-82.06) |
| VEGF | 0.81 (0.70-0.91) | <0.001 | 38 | 93.55 (78.58-99.21) | 55.17 (35.69-73.55) |

95% CI = 95% confidence interval.
