# Supplementary material for: Combined Analysis of IFN-γ, IL-2, IL-5, IL-10, IL-1RA and MCP-1 in QFT Supernatant Is Useful for Distinguishing Active Tuberculosis from Latent Infection
Source: PLoS One. 2016 Apr 1;11(4):e0152483. doi: 10.1371/journal.pone.0152483 (PMC4817970; doi:10.1371/journal.pone.0152483)
Supplement: S3 Table — (DOCX) [file pone.0152483.s005.docx]

S3 Table. Correlations between concentrations of cytokines and clinical findings (Nil).

Pearson’s correlation coefficients were also calculated to evaluate the relationships between the concentration of the cytokines in all active TB patients and several clinical variables (age, white blood cells, neutrophils, lymphocytes, monocytes, albumin, C-reactive protein (CRP) and erythrocyte sedimentation rate (ESR)).

|  | Correlation coefficient (*p*-value) | | | | | |
| --- | --- | --- | --- | --- | --- | --- |
|  | IL-10 | IFN-γ | MCP-1 | IL-1RA | IL-15 | IL-12 |
| Age (y) | 0.1863  (0.32) | 0.10  (0.60) | -0.15  (0.41) | 0.00  (0.99) | 0.25  (0.17) | 0.22  (0.23) |
| WBC (/μL) | -0.1045  (0.58) | -0.06  (0.74) | -0.46  (0.01) | -0.23  (0.21) | -0.34  (0.06) | -0.10  (0.59) |
| Neutrophil (/μL) | -0.0715  (0.70) | -0.09  (0.62) | -0.44  (0.01) | -0.19  (0.30) | -0.28  (0.13) | -0.08  (0.68) |
| Lymphocyte (/μL) | -0.2732  (0.14) | -0.03  (0.86) | -0.45  (0.01) | -0.31  (0.09) | -0.51  (0.004) | -0.21  (0.25) |
| Monocyte (/μL) | -0.0187  (0.92) | 0.21  (0.25) | -0.22  (0.23) | -0.09  (0.63) | -0.13  (0.47) | -0.07  (0.72) |
| Albumin (g/dL) | -0.2467  (0.18) | 0.00  (0.99) | 0.25  (0.18) | -0.44  (0.01) | -0.31  (0.09) | -0.26  (0.17) |
| CRP (mg/dL) | 0.1655  (0.37) | 0.25  (0.18) | -0.22  (0.23) | 0.23  (0.22) | 0.28  (0.12) | 0.34  (0.06) |
| ESR (mm/hr) | 0.3447  (0.11) | 0.06  (0.80) | -0.43  (0.04) | 0.35  (0.10) | 0.15  (0.50) | 0.36  (0.09) |

WBC, white blood cells; CRP, C-reactive protein; ESR, erythrocyte sedimentation rate; CXR, chest X-ray.
